# Prognostic Value of CTA-Derived Left Ventricular Mass in Neonates with Congenital Heart Disease

**DOI:** 10.3390/diagnostics11071215

**Published:** 2021-07-06

**Authors:** Stephan Ellmann, Julie-Marie Nickel, Rafael Heiss, Nouhayla El Amrani, Wolfgang Wüst, Oliver Rompel, Andre Rueffer, Robert Cesnjevar, Sven Dittrich, Michael Uder, Matthias S. May

**Affiliations:** 1 Department of Radiology, University Hospital Erlangen, Maximiliansplatz 3, 91054 Erlangen, Germany; juliemarienickel@gmail.com (J.-M.N.); rafael.heiss@uk-erlangen.de (R.H.); elamrani.nouhayla@gmail.com (N.E.A.); oliver.rompel@uk-erlangen.de (O.R.); michael.uder@uk-erlangen.de (M.U.); matthias.may@uk-erlangen.de (M.S.M.); 2 Imaging Science Institute Erlangen, Ulmenweg 18, 91054 Erlangen, Germany; wolfgang.wuest@martha-maria.de; 3 Department of Radiology, Martha Maria Hospital Nuremberg, 90491 Nuremberg, Germany; 4 Department of Pediatric Cardiac Surgery, University Hospital Hamburg Eppendorf, 20246 Hamburg, Germany; a.rueffer@uke.de; 5 Department of Pediatric Cardiac Surgery, University Hospital Erlangen, Loschgestraße 15, 91054 Erlangen, Germany; robert.cesnjevar@uk-erlangen.de; 6 Department of Pediatric Cardiology, University Hospital Erlangen, Loschgestraße 15, 91054 Erlangen, Germany; sven.dittrich@uk-erlangen.de

**Keywords:** congenital heart disease, hypoplastic left heart, computed tomography angiography, cardiac computed tomography, heart surgery, heart imaging

## Abstract

For therapeutic decisions regarding uni- or biventricular surgical repair in congenital heart disease (CHD), left ventricular mass (LVM) is an important factor. The aim of this retrospective study was to determine the LVM of infants with CHD in thoracic computed tomography angiographies (CTAs) and to evaluate its usefulness as a prognostic parameter, with special attention paid to hypoplastic left heart (HLH) patients. Manual segmentation of the left ventricular endo- and epicardial volumes was performed in CTAs of 132 infants. LVMs were determined from these volumes and normalized to body surface area. LVMs of patients with different types of CHD were compared to each other using analyses of variances (ANOVA). An LVM cutoff for discrimination between uni- and biventricular repair was determined using receiver operating characteristics. Survival rates were calculated using Kaplan–Meier statistics. Patients with a clinical diagnosis of an HLH had significantly lower mean LVM (21.88 g/m^2^) compared to patients without applicable disease (50.22 g/m^2^; *p* < 0.0001) and compared to other CHDs, including persistent truncus arteriosus, left ventricular outflow tract obstruction, transposition of the great arteries, pulmonary artery stenosis or atresia, and double-outlet right ventricle (all, *p* < 0.05). The LVM cutoff for uni- vs. biventricular surgery was 33.9 g/m^2^ (sensitivity: 82.3%; specificity: 73.7%; PPV: 94.9%). In a subanalysis of HLH patients, a sensitivity of 50.0%, specificity of 100%, PPV of 100%, and NPV of 83.3% was determined. Patient survival was not significantly different between the surgical approaches or between patients with LVM above or below the cutoff. LVM can be measured in chest CTA of newborns with CHD and can be used as a prognostic factor.

## 1. Introduction

Congenital heart disease (CHD) is the most common congenital anomaly, affecting 4–10 out of 10,000 live births [1]. Those defects can either be managed palliatively with a single-ventricle correction or repaired in a biventricular manner. Factors influencing this decision include ventricle size, valve anatomy, complexity of intracardiac anatomy, feasibility of conduit placement, patient age, and surgical skills [2,3]. In the case of single-ventricle palliation, patients in most cases receive a bidirectional Glenn shunt followed by the Fontan operation. This procedure exhibits certain disadvantages compared to biventricular repair [4], which at least in theory maintains normal physiology and leads to better exercise tolerance, fewer arrhythmias, and better long-term outcomes [5]. Whereas in adult patients, left ventricular mass is associated with increased cardiovascular morbidity and mortality [6,7,8], ventricular size is one of the most important features for the decision towards uni- or biventricular repair in infant CHD patients [9]. After biventricular repair, both the right and left ventricle have to be able to maintain pulmonary and systemic circulation, respectively. Certain guidelines have been published pointing to the minimal size required for the left ventricle to maintain systemic circulation [10,11,12].

In CHD diagnosis and evaluation, transthoracic echocardiography is considered the gold standard in the determination of ventricular masses and further anatomic features to guide surgical decisions, but it is operator-dependent. Moreover, in case of complex anomalies, large septal defects, or partly retrosternal structures, echocardiography may feature drawbacks. In these cases, and for three-dimensional evaluations of the great vessels, computed tomography (CT) is increasingly being used for diagnostic imaging purposes. Conventional single-source CT is limited in accelerating table feeds because of the gaps occurring in the acquired volume. Second- and third-generation dual-source CT systems provide a high-pitch spiral mode. Those CT systems offer the highest table feeds at pitch values above 3.0, as their two detectors mounted with an offset of 90 degrees are able to complement each other’s gaps. With high pitches and fast table speeds, entire volumetric data acquisition can be obtained within one single cardiac cycle [13,14]. Compared to ECG-gated sequential scans, high-pitch acquisitions feature considerably lower radiation exposure [15], which is of particular importance for pediatric patients.

Several indications for cardiac CT, including evaluation of the coronary anatomy and aortic anomalies, detection of major aortopulmonary collateral arteries, and the presurgical workup in complex cases, have been established in the past [5,16,17,18,19]. This retrospective study aimed to additionally determine the LVM of patients that underwent CT angiographies (CTAs) of the chest and cluster them by the reported clinical diagnoses. Surgical strategies have been taken from the medical records to calculate an LVM cutoff for uni- or biventricular surgical approaches.

## 2. Materials and Methods

### 2.1. Patients

This retrospective single-center study was approved by the institutional review board and complies with the declaration of Helsinki. Patients suspected for or already diagnosed with complex CHD that underwent free-breathing CTA of the chest within a time period of 5 years were included (2010–2015; n = 132). All CT examinations were clinically indicated to further define the extent of CHD or to facilitate sufficient planning of subsequent surgical procedures. The investigated CHDs included persistent truncus arteriosus (PTA), tetralogy of Fallot (TOF), left ventricular outflow tract obstruction (LVOTO), hypoplastic left heart (HLH), right ventricular outflow tract obstruction (RVOTO), double-outlet right ventricle (DORV), transposition of the great arteries (TGA), pulmonary stenosis or atresia (PS/PA), thoracic aortic pathology (TAP, including stenosis and atresia), patent ductus arteriosus (PDA), and septal defect (SD).

### 2.2. Computed Tomography

Imaging was performed using second- or third-generation dual-source computed tomography (Siemens CT Somatom Definition Flash or Somatom Force, Siemens Healthineers, Forchheim, Germany) with lowest tube voltages of either 80 kV or 70 kV. Reference tube current for anatomy-based automatic tube current modulation (CareDose 4D) was set to 400 mAs per rotation. Acquisition collimation was set to 2 × 128 × 0.6 mm or 2 × 192 × 0.6 mm using a z-flying focal spot. Scans were performed with a prospectively ECG-triggered high-pitch (3.2 and 3.4) spiral acquisition mode (Flash Spiral Cardio mode, Siemens Healthineers, Forchheim, Germany), triggered to the systolic phase. Premedication was used for patients older than 4 months (0.1 mg/kg Midazolam). In case of ongoing agitation, a dose of 0.3 mg/kg ketamine S was applied directly in the scanner room. Patients below the age of 4 months were examined without sedation. Oral or intravenous beta-blockers were not administered, as their use in CHD patients is potentially contraindicated, insufficiently effective, or even harmful [20]. Two milliliters of iodinated contrast agent per kg body weight (Iomeron 350, Bracco, Konstanz, Germany) was used. If the resulting volume was <10 mL, the contrast agent was diluted in 0.9% NaCl solution to a total volume of 10 mL prior to injection into a peripheral vein over a time period of 5–10 s. Manual or mechanical (Accutron CT-D, Medtron GmbH, Saarloius, Germany) injection was chosen depending on the site, size, and safety of vascular access. The diagnostic scan was performed in craniocaudal direction from the upper thoracic aperture to the posterior costodiaphragmatic angle without further delay. Images were reconstructed (weighted filtered back projection) in the minimum slice thickness with an overlapping increment (0.6/0.5 mm) using a dedicated vascular soft tissue kernel (B26f) and transferred to the institutional picture archiving and communication system and to a 3D postprocessing workstation (syngo.via, Siemens Healthineers, Forchheim, Germany). Radiation exposure was assessed as volumetric CT dose index (CTDIvol), referring to a 32 cm acrylic phantom, and dose length product (DLP). Effective dose (ED) was assumed as DLP × *k*, using an individual, linear interpolation of the conversion factors reported in the literature [21] for chest CT at 80 kV between neonates (*k_I_* = 0.0823 mSv/mGy × cm) and 1-year-old children (*k_II_* = 0.0525 mSv/mGy × cm) as a function of days of life (*d*) by the following Equation (1):

(1) $$ED=DLP\times \left(k_{II}+\;\frac{d\times \left(k_{I}-k_{II}\right)}{365}\right)$$

### 2.3. Left Ventricular Mass

**Figure 1:**
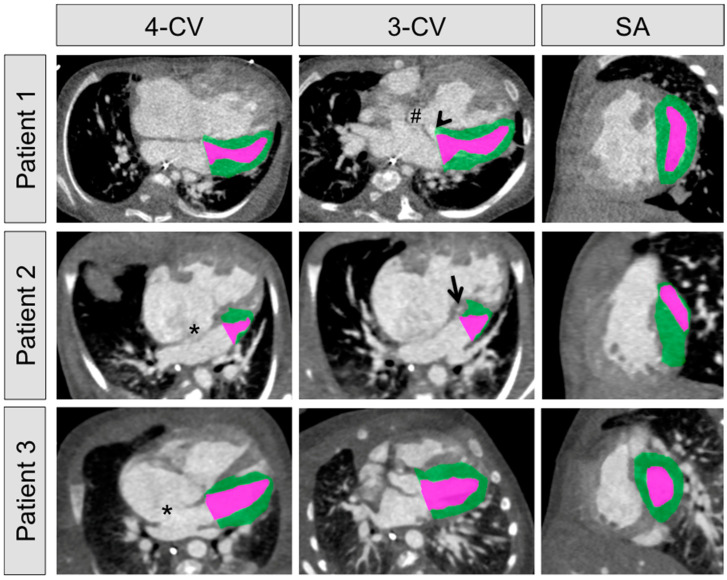
Exemplary segmentation of three HLH patients’ left ventricular volumes, with the endocardial volumes depicted in violet and the epicardial volumes depicted in green. The panel shows four-chamber views (4-CV; first column), three-chamber views (3-CV; middle column), and short-axis views (SA; right column). Patient 1: The 98-days-old female patient featured an HLH along with a ventricular septal defect (arrowhead), a double-outlet right ventricle (#), a left ventricular outflow tract obstruction, and an aortic coarctation. Segmentation yielded an epicardial volume of 15.48 cm^3^ and an endocardial volume of 4.29 cm^3^. Subtraction yielded a left ventricular muscle volume of 11.19 cm^3^. The corresponding left ventricular mass was 41.1 g/m^2^. The patient received a biventricular repair, consisting of a correction of the double-outlet right ventricle, along with a reconstruction of both the mitral and the tricuspidal valve at the age of 2.5 months. Patient 2: The 2-days-old male patient featured an HLH along with an atrial septal defect (*), an aortic coarctation, a patent ductus arteriosus, and a left ventricular outflow tract obstruction (arrow). Segmentation yielded an epicardial volume of 2.63 cm^3^ and an endocardial volume of 0.77 cm^3^. Subtraction yielded a left ventricular muscle volume of 1.86 cm^3^. The corresponding left ventricular mass was 9.3 g/m^2^. This patient received a Norwood procedure at the age of 7 days and a Fontan completion at the age of 3.5 years. Patient 3: The 4-days-old female patient suffered from a t(1q; 14q) translocation and featured a borderline HLH along with an atrial septal defect (*), an aortic coarctation, and a patent ductus arteriosus. Segmentation yielded an epicardial volume of 7.36 cm^3^ and an endocardial volume of 2.09 cm^3^. Subtraction yielded a left ventricular muscle volume of 5.27 cm^3^. The corresponding left ventricular mass was 28.9 g/m^2^. The patient received a biventricular repair, consisting of a repair of the aortic coarctation and a septal defect patch closure at the age of 10 days.

All measurements were performed in analogy to the international recommendations for chamber quantification in echocardiography [22]. Multiplanar reformations were used to obtain short- and long-axis planes corresponding to those typically used in echocardiography. Left ventricular volume was assessed by manual segmentation of the left ventricular endo- and epicardial volumes and subsequent subtraction. LVM was calculated by multiplication of the left ventricular muscle volume and the specific gravity constant of heart muscle (1.05 g/mL) [23]. Values were normalized to body surface area following the equation proposed by Mosteller et al. [24]. Figure 1 depicts the segmentation of three exemplary patients’ left hearts. LVMs were assessed by two board-certified radiologists (M.S.M. and O.R.), each with >10 years of experience in infant cardiac radiology.

### 2.4. Surgery

The medical and surgical strategies for CHD treatment were discussed for each patient separately in a weekly institutional interdisciplinary CHD board. Consensus-driven decisions were based on clinical presentation, medical history, multimodality imaging, and social background. Surgical treatment was retrospectively noted from the archives. December 2019 or death was defined as an endpoint for the survival analysis.

### 2.5. Statistics

Statistical calculations were performed using RStudio (RStudio, Inc., Boston, MA, USA) and GraphPad Prism 6 (GraphPad Software, La Jolla, CA, USA). The significance of differences between the normalized LVMs of the patients was calculated using nonparametric Kruskal–Wallis tests based on analyses of variances (ANOVA), followed by Dunn’s tests for multiple comparisons. *p* values < 0.05 were considered statistically significant. Confidence intervals (CIs) were calculated at a confidence level of 95%. UpSet was used to analyze intersections and aggregates of intersections between the different diseases [25]. A receiver operating characteristic (ROC) analysis was performed to determine an LVM cutoff for discrimination between uni- or biventricular surgery. Pythagoras’s theorem was used to identify the cutoff value on the ROC curve with closest approximation to the diagram’s top left corner. Cross tables were used to estimate additional measures of diagnostic accuracy (positive and negative predictive values (PPVs and NPVs, respectively) and overall accuracy). Kaplan–Meier curves were calculated for survival analyses. For the analysis of the subgroup of HLH patients, the same statistical methods, tests, and thresholds were applied.

## 3. Results

### 3.1. Patients

**Figure 2:**
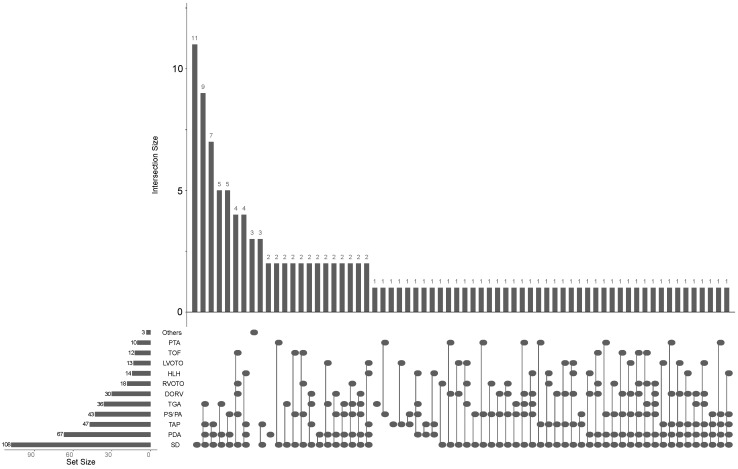
Intersection scheme of congenital heart defects in 132 patients: persistent truncus arteriosus (PTA), tetralogy of Fallot (TOF), left ventricular outflow tract obstruction (LVOTO), hypoplastic left heart (HLH), right ventricular outflow tract obstruction (RVOTO), double-outlet right ventricle (DORV), transposition of the great arteries (TGA), pulmonary stenosis or atresia (PS/PA), thoracic aortic pathology (TAP), persistent ductus arteriosus (PDA), and septal defect (SD; n = 18 isolated atrial SDs; n = 18 isolated ventricular SDs; n = 72 atrioventricular SDs). Black dots indicate the presence of a given disease or disease combination. Horizontal bar diagrams indicate the number of the single diseases observed (set size), and vertical bar diagrams indicate the numbers of the observed disease combinations (intersection size).

Median patient age was 9 days (range, 1–320 days), median size was 51.5 cm (range, 38–73 cm), median weight was 3.5 kg (range, 1.6–8.5 kg), and median body mass index was 13.5 kg/m^2^ (range, 10.0–20.2 kg/m^2^). In 11 out of the 132 investigated patients, no applicable disease (NAD) was observed. Those 11 patients turned out to suffer from pneumonia (n = 3), a pulmonary sling (n = 2), a double aortic arch (n = 1), pulmonary heart disease (n = 1), Kawasaki disease (n = 1), or from an inspiratory stridor that was, however, not caused by a pulmonary sling or double aortic arch (n = 3). Among the remaining 121 patients, only a minority had an isolated CHD: 11 patients suffered from an isolated SD, 2 patients from an isolated PDA, 1 from an isolated TAP, and 1 from an isolated TGA. Three patients (“others”) were not classifiable within the scheme of CHD (one dilatative cardiomyopathy, one right aortic arch without stenosis, and one sequester of the pulmonary segments 8–10). A majority of patients suffered from combinations of different CHDs, with an SD present in 108 patients (81.8%), followed by PDA (n = 67, 50.7%) and TAP (n = 47, 35.6%). TGA and DORV malpositions of the great arteries, were found in 36 and 30 patients, respectively (27.3% and 22.7%), and a TOF was found in 12 patients (9.1%). Ten patients had a PTA (7.6%). All patients that were considered HLH (n = 14) had additional defects accounting for a right to left shunt (SD or PDA). Most frequently, HLH was associated with TAP (n = 11). DORV was associated with HLH in four patients and TGA in two patients. Combinations of HLH with outflow-tract obstructions were present in three patients (LVOTO) and one patient (RVOTO). A schematic representation of the different CHDs and their associations is presented in Figure 2.

### 3.2. Computed Tomography

The median anatomically adapted tube current–time product was 76 mAs per rotation (range, 14–264 mAs). Over all examinations, median CTDIvol was 0.36 mGy (range, 0.11–2.07 mGy) and the median DLP was 6 mGy × cm (range, 1.7–45 mGy × cm). The resulting effective dose was 0.32 mSv (range, 0.089–3.38 mSv). The patients’ heart rate averaged 137 ± 24 beats/minute (95% CI: 132–141 beats/minute). The mean heart phase averaged 47.0 ± 15.9% (95% CI: 44.1–49.9%).

### 3.3. Left Ventricular Mass

**Figure 3:**
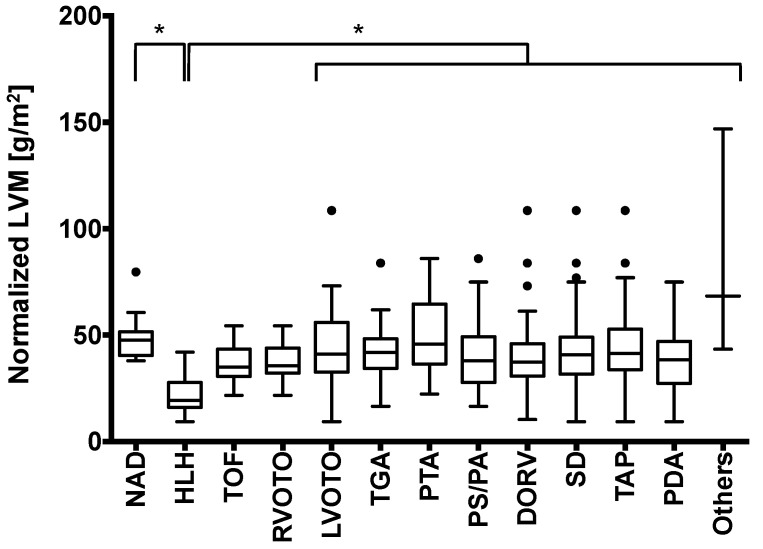
Boxplot of the left ventricular masses (LVMs) normalized to body surface area clustered by the different malformations: No applicable disease (NAD, n = 11), hypoplastic left heart (HLH, n = 14), tetralogy of Fallot (TOF, n = 12), left ventricular outflow tract obstruction (LVOTO, n = 13), right ventricular outflow tract obstruction (RVOTO, n = 18), transposition of the great arteries (TGA, n = 36), persistent truncus arteriosus (PTA, n = 10), pulmonary stenosis or atresia (PS/PA, n = 43), double-outlet right ventricle (DORV, n = 30), septal defect (SD, n = 108), thoracic aortic pathology (TAP, n = 47), persistent ductus arteriosus (PDA, n = 67), and others (n = 3). Significant differences are marked with asterisks. For a detailed listing of the respective *p* values, see Table S1. The LVM of HLH patients was significantly reduced compared to all other subgroups except TOF and RVOTO. Boxplots follow the Tukey definition, with whiskers indicating the 1.5-fold interquartile range. Outliers are marked as black dots.

Significant differences regarding LVM were found between the subgroups by Kruskal–Wallis analysis (*p* < 0.0001). Highest LVMs among all patients suffering from CHD were observed for the PTA patients (49.34 ± 18.9 g/m^2^; 95% CI: 35.1–63.6 g/m^2^), followed by LVOTO (45.20 ± 24.1 g/m^2^; 95% CI: 30.1–60.4 g/m^2^) and TAP patients (42.76 ± 18.3 g/m^2^; 95% CI: 37.3–48.2 g/m^2^). Lowest LVMs were observed for HLH (21.88 ± 9.90 g/m^2^; 95% CI: 16.2–27.6 g/m^2^), followed by patients suffering from TOF (36.93 ± 9.4 g/m^2^; 95% CI: 30.7–43.2 g/m^2^). Under statistical considerations, only the LVMs of HLH patients (21.88 ± 9.90 g/m^2^) differed significantly from those of NAD patients (50.22 ± 11.24 g/m^2^; 95% CI: 42.3–58.1 g/m^2^; Figure 3; *p* < 0.0001) in the post hoc test, with HLH patients featuring significantly reduced LVM. The LVMs of HLH patients were moreover significantly lower compared to all other CHD except RVOTO and TOF (all, *p* < 0.05). All other analyzed pathologies featured no significant differences compared to NAD patients and in all pairwise comparisons (all, *p* > 0.44; Table S1).

### 3.4. Surgery

**Figure 4:**
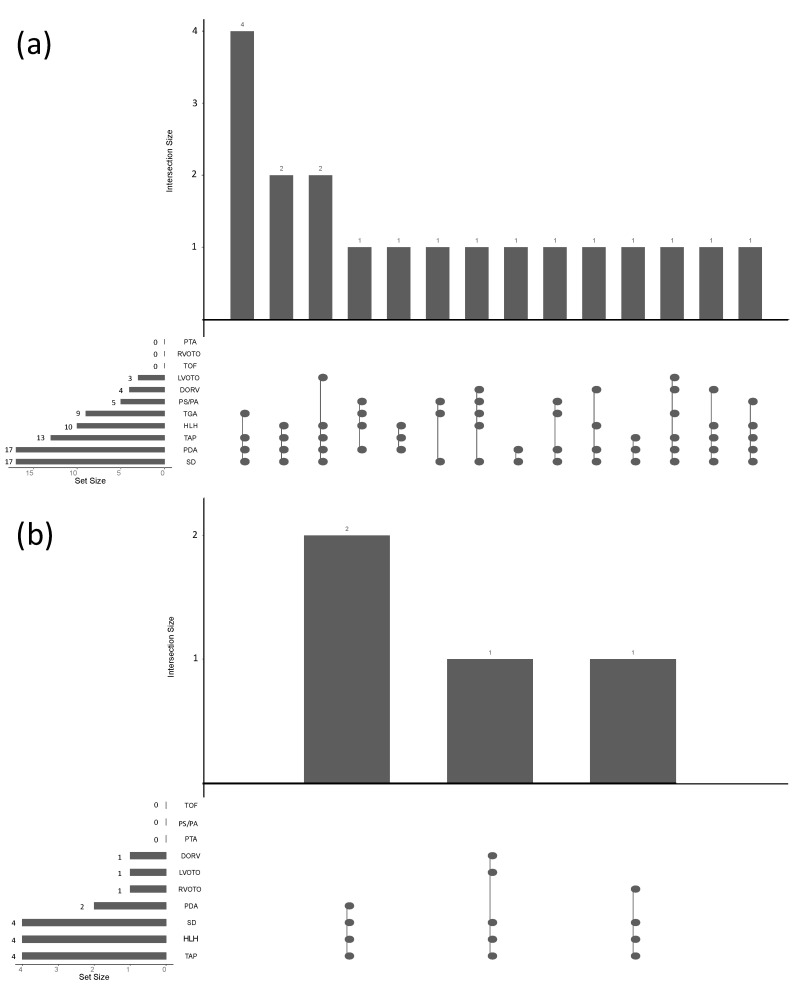
(**a**) Intersection scheme of patients having received univentricular repair (n = 19). Black dots indicate the presence of a given disease or disease combination: persistent truncus arteriosus (PTA), right ventricular outflow tract obstruction (RVOTO), tetralogy of Fallot (TOF), left ventricular outflow tract obstruction (LVOTO), double-outlet right ventricle (DORV), pulmonary stenosis or atresia (PS/PA), transposition of the great arteries (TGA), hypoplastic left heart (HLH), thoracic aortic pathology (TAP), persistent ductus arteriosus (PDA), and septal defect (SD). Horizontal bar diagrams indicate the number of the single diseases observed (set size), and vertical bar diagrams indicate the numbers of the observed disease combinations (intersection size). Note: One patient featured an SD (which was a large inlet ventricular SD), along with a PDA and a TAP. This patient was initially treated with a univentricular approach but received a biventricular repair at the age of 3 years. A second patient with an SD and a PDA also received a univentricular approach but died 1 month after the surgery, before further surgical strategies were discussed in detail. (**b**) Four patients with HLH received biventricular repair. All those patients featured SD and TAP. One patient additionally exhibited a combination with a DORV and should thus be regarded as a hypoplastic left heart-related malformation patient; the remaining three patients were true hypoplastic left heart syndrome patients.

On average, surgery was performed 24 days after the CT examination. Of the 132 patients included in this study, 19 underwent univentricular palliative surgery and 113 underwent biventricular repair. In patients with univentricular palliation, none of the underlying defects were isolated, but part of a combination of 2–6 malformations (Figure 4a). The majority of cases with a univentricular approach were HLH patients (n = 10; 53%). Other cases were either TGAs (n = 7; 37%) or large atrioventricular SDs (n = 2; 10%).

Regarding the HLH patients, 10 out of 14 underwent univentricular repair (71%; mean LVM, 17.3 ± 5.2 g/m^2^; 95% CI: 13.6–21.0 g/m^2^), whereas n = 4 were repaired biventricularly (28.6%; mean LVM, 33.2 ± 10.1 g/m^2^; 95% CI: 17.3–49.3 g/m^2^). All HLH patients that underwent a biventricular approach had an associated TAP, and only one of them had an additional malposition (DORV; 25%). Outflow tract obstructions were present in 50% of the patients. No PS/PA was present in these patients (Figure 4b). HLH patients that underwent univentricular surgery were more likely to exhibit a malposition (2 × TGA, 2 × DORV; 40%) and obstructions of the outflow tracts or PS/PA (70%).

**Figure 5:**
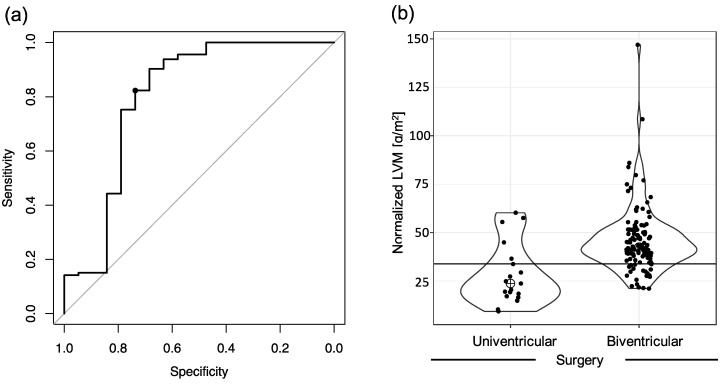
(**a**) ROC curve to determine an optimal left ventricular mass (LVM) cutoff for uni- vs. biventricular surgery. The black circle represents the curve’s closest approximation to the diagram’s top left corner (33.9 g/m^2^; sensitivity, 82.3%; specificity, 73.7%). The area under the ROC curve was 0.801 (95% confidence interval, 0.655–0.946). (**b**) Violin plot comparing the normalized LVM of patients having received uni- and biventricular repair, along with black dots indicating the patients’ individual values. The horizontal black line marks the optimal cutoff value of 33.9 g/m^2^.

Using ROC analysis, an LVM cutoff of 33.9 g/m^2^ featuring a sensitivity of 82.3% (95% CI: 74.0–88.8%) and specificity of 73.7% (95% CI: 48.8–90.9%) was determined (Figure 5a). The area under the ROC curve was 0.801 (95% CI: 0.655–0.946). The corresponding PPV was 94.9% (95% CI: 88.5–98.3%). Five out of the nineteen patients that underwent univentricular repair had an LVM above this cutoff (26%; Figure 5b). Only 20 patients with an LVM below the cutoff were treated with a biventricular approach (18%; Figure 5b). Two out of the four HLH patients that underwent biventricular repair had an LVM above the cutoff, and no HLH patient above the cutoff received univentricular palliation, resulting in a sensitivity of 50.0%, specificity of 100%, PPV of 100%, and NPV of 83.3% for the established cutoff within the population of HLH patients (Table 1; for detailed cross tables see Table S2).

**Figure 6:**
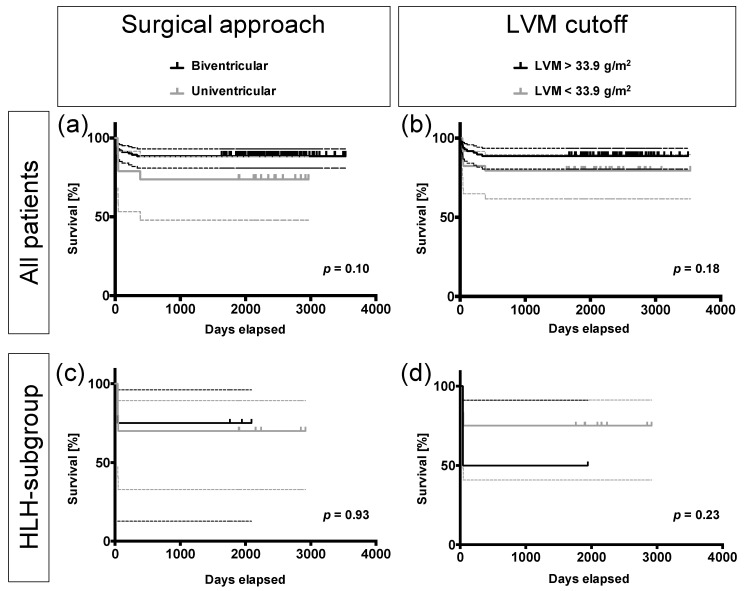
Kaplan–Meier analysis. (**a**) No significant survival differences were observed between the uni- and biventricular subgroup (*p* = 0.10) or (**b**) between patients with left ventricular masses above or below the cutoff of 33.9 g/m^2^ (*p* = 0.18). (**c**) In an analysis of the HLH subgroup, no significant differences in survival were observed regarding the surgical approach (*p* = 0.93) or (**d**) the patients’ LVM with respect to the determined cutoff of 33.9 g/m^2^ (*p* = 0.23). Dotted lines depict the 95% confidence intervals of the respective survival curves.

Mean follow-up time of all patients was 2395 ± 450 days (6.5 years). Overall 5-year survival was 86.4%. Patients that underwent univentricular surgery had a lower survival (73.7%) compared to patients having received a biventricular approach (88.4%), but this difference was not significant (*p* = 0.10; Figure 6a). Patients with an LVM below the determined cutoff had a slightly lower survival (79.4%) compared to patients with an LVM above (88.7%), however without statistically significant differences regardless of the type of surgery (*p* = 0.18; Figure 6b). Of note, all patients having survived the first year after surgery also managed the first 5 years.

With respect to the subgroup of HLH patients, no significant differences in survival were observed either. HLH patients with univentricular palliation had a survival of 70%, whereas biventricularly treated patients had a survival of 75% (*p* = 0.93; Figure 6c). HLH patients with an LVM above the cutoff of 33.9 g/m^2^ had a survival of 50%, whereas HLH patients with an LVM below the cutoff had a survival of 75% (*p* = 0.23; Figure 6d). As the HLH subgroup consisted of only 14 patients, the respective confidence intervals, however, were rather wide.

## 4. Discussion

This study demonstrates that the normalized LVM in neonates suffering from CHD can be calculated from cardiac CT and proposes an LVM cutoff of 33.9 g/m^2^ to assist the decision towards uni- or biventricular repair. It is particularly noteworthy that the results of this study are based on a measurement of only the residual left ventricle. The finding that the left residual ventricle contributes to the outcome of HLH patients has not been published so far and deserves critical appraisal. The provided LVM cutoff, in addition, provides prognostic estimates for the survival of the patients, though we did not observe significant differences in survival between the surgical approaches or between the groups of patients above or below the LVM threshold. Clinical diagnosis of HLH was the only clustered subgroup that had a significantly reduced LVM compared to non-CHD patients and compared to all other CHDs except TOF and RVOTO. However, stratifying the heterogeneous combinations of CHD anomalies into uni- or biventricular repair recommendations definitely requires multiparametric approaches, with the LVM representing only one aspect. In particular, some types of unbalanced atrioventricular septal defects, DORV [26,27,28] or TGA combined with VSD and PS [5], remain challenging. In TGA patients, LVM can be considered a prognostic parameter. In a study from 2001 [29], the indication for a left ventricular retraining in TGA patients was analyzed, and an inferior limit of 35 g/m^2^ was determined as a “safe landmark”. Interestingly, this value is quite similar to the threshold proposed in our study. However, the authors stated that this value was quite arbitrary and subject to further discussion.

Moreover, in addition to the LVM, accompanying pathologies and the component make-up of the chambers within the ventricles have to be taken into account [9]. Particularly in those complex cases, CTA complements echocardiography in the presurgical evaluation by excellent visualization of the great arteries and coronaries and the possibility to three-dimensionally evaluate the overall picture of the defects.

In the past, several criteria have already been published to assist in the conscientious individual decision to perform a biventricular repair or single ventricle palliation [30]. Rhodes et al. developed a scoring system based on body surface area, indexed aortic root dimension, left ventricular long-axis dimension, and indexed mitral valve area to predict in-hospital mortality of patients with aortic stenosis after biventricular repair [10]. This scoring system, however, is not applicable to HLH patients without valvular malformations, as those patients commonly perform better than predicted by traditional scoring methods [16,17,18]. The echocardiographic evaluation in these patients is, moreover, challenging because of the distorted anatomy and difficult endocardial delineation [31]. However, in addition to the proposed scoring system, Rhodes et al. name an LVM < 35 g/m^2^ as one of four risk factors for increased in-hospital mortality [10]. Kim et al. already used pediatric cardiac CT in one single case report to evaluate left ventricular volume index for the multiparametric surgical decision and concluded that this method “has great potential to provide an accurate measurement of LV volume, which is essential for determining the optimal surgical repair thereby improving the short- and long-term surgical outcome in patients with congenital heart disease and a marginally small LV” [19].

Though centered on LVM, our study substantiates these claims and provides a precise cutoff value that could be used as an additional quantitative parameter for the individual surgical decision, especially within the scope of interdisciplinary boards. Survival rates from our HLH collective for univentricular palliation (70%) were comparable to the range reported in the large multicenter single ventricle reconstruction trial (3 years, 61–67%; 6 years, 59–64%) [32,33], regardless of the surgical Norwood I approach. Moreover, we were able to show that the LVM obtained from CTA in HLH can help to estimate the outcome of the patients. Of note, according to the European Congenital Heart Surgeons Association (ECHSA) database [34], the 5-year survival of HLH patients in our institution is 84.7%, and thus 10–15% higher than the survival rate determined for HLH patients of our study cohort. However, during the recruitment period, only 14 HLH patients received cardiac CT, but 51 HLH patients underwent surgery. It thus has to be assumed that the cohort of HLH patients having received cardiac CT imaging represents a rather high-risk group with an inferior prognosis, whereas low-risk HLH patients with a better prognosis received cardiac CT less frequently. This would lead to an overrepresentation of high-risk HLH patients in our study cohort and explain the discrepant survival rates.

Some limitations of our study merit consideration: Underdevelopment of the left heart can be associated with a broad spectrum of concomitant defects. One rather strict definition is the hypoplastic left heart syndrome as a hypoplasia of the entire left heart: hypoplastic left ventricle plus atresia, stenosis, or hypoplasia of the aortic and/or mitral valve, plus hypoplasia of the ascending aorta and aortic arch, but normally aligned great arteries without a common atrioventricular junction [35]. Other pathologies are more likely termed hypoplastic left heart-related malformations in the literature. We did not differentiate between these different classifications for this study because of the large differences within each subcategory and therefore unpredictable impact on the surgical decision and outcome. Moreover, the sizes of subgroups would have been further shrunk with additional subdivisions. For the sake of completeness, five of our HLH patients could be considered as HLHM because of their combination with DORV and/or TGA, and the remaining nine patients would rather have fitted into the HLHS classification (Figure 2).

Moreover, in CHD, every single patient is unique regarding the combination, extent, and characteristics of malformations. Surgical decisions in all patients were based on multiparametric approaches including not only anatomy and pathophysiology, but also social and socioeconomic factors, as well as parents’ preferences. As every patient as an individual lacks their own negative control, there is no way to determine if one or another patient might have potentially done better with the alternative surgical approach.

In addition, this single-center study is unable to offer comparisons between different surgical philosophies, which are linked to the equipment, abilities, and preferences of every site in this highly specialized medical field. Novel strategies, such as hybrid approaches and staged left ventricular recruitment, further complicate the decision-making and also depend on institutional experience [36,37].

To continue on limitations, no inter-reader agreement was determined between the radiologists assessing the LVM. Moreover, the number of patients included in this study is relatively high, but subgroups may only have small sample sizes. However, in relation to the overall low incidence of CHD, the relatively long follow-up of over 5 years, the novelty of the applied technology, and the patient numbers of other studies in the literature, our results should be of value and contribute to future clinical CHD workup.

Last, the patients included in this study were scanned at rather high heart rates for clinical reasons, as beta-blockers are potentially contraindicated in CHD patients [20]. Triggering the scan to the systolic phase might have introduced a certain bias regarding the LVM quantification, as the specific gravity constant for heart muscle is calibrated to the end-diastolic phase [38,39]. However, this gravity constant is subject to various variabilities [40], so that the value of 1.05 g/mL used in this study represents only a certain approximation anyway.

## 5. Conclusions

To conclude, CTA of the chest allows for measuring the LVM in neonates. HLH patients suffer from significantly smaller LVM in comparison to patients without CHD and in comparison to all other types of CHD except RVOTO and TOF. Quantification of the LVM in CHD patients provides a new parameter for an interdisciplinary, multiparametric surgical workup. The LVM cutoff of 33.9 g/m^2^ may aid the decision towards uni- or biventricular repair and help to estimate patient survival.

## Figures and Tables

**Table 1:** Diagnostic accuracy estimates along with 95% confidence intervals for the entire patient population and the subgroup of HLH patients.

	All Patients		HLH Subgroup	
	Estimation	95% CI	Estimation	95% CI
Sensitivity	82.3%	74.0–88.8%	50.0%	6.8–93.2%
Specificity	73.7%	48.8–90.9%	100%	58.7–100%
PPV	94.9%	88.5–98.3%	100%	9.4–100%
NPV	41.2%	24.6–59.3%	83.3%	51.6–97.9%
Overall accuracy	81.1%	73.3–87.4%	85.7%	57.2–98.2%

The table lists sensitivities, specificities, positive and negative predictive values (PPVs and NPVs, respectively), and overall accuracy along with 95% confidence intervals (CIs) for the entire patient population and the subgroup of HLH patients for uni- vs. biventricular surgery when applying an LVM cutoff of 33.9 g/m^2^.

## Data Availability

An anonymized data set of the raw data used for this study can be found at DOI 10.17605/OSF.IO/K86N9.

## Supplementary Materials

The following are available online at https://www.mdpi.com/article/10.3390/diagnostics11071215/s1, Table S1: Multiple comparisons of LVM with respect to different groups of congenital heart diseases. Table S2: Diagnostic cross tables.
