# Supplementary material for: Prognostic Value of CTA-Derived Left Ventricular Mass in Neonates with Congenital Heart Disease
Source: Diagnostics (Basel). 2021 Jul 6;11(7):1215. doi: 10.3390/diagnostics11071215 (PMC8303678; doi:10.3390/diagnostics11071215)
Supplement: Supplementary file 1 [file diagnostics-11-01215-s001.zip › diagnostics-1258709-supplementary.pdf]

Supplementary Table S1: Multiple comparisons of LVM with respect to different groups of congenital heart diseases.

Dunn's multiple comparisons test	Adjusted p Value
NAD vs. HLH	< 0.0001
NAD vs. TOF	> 0.9999
NAD vs. LVOTO	> 0.9999
NAD vs. RVOTO	0.9404
NAD vs. TGA	> 0.9999
NAD vs. PTA	> 0.9999
NAD vs. PS/PA	> 0.9999
NAD vs. DORV	> 0.9999
NAD vs. SD	> 0.9999
NAD vs. TAP	> 0.9999
NAD vs. PDA	0.4467
NAD vs. Others	> 0.9999
HLH vs. TOF	> 0.9999
HLH vs. LVOTO	0.0454
HLH vs. RVOTO	0.3385
HLH vs. TGA	0.0010
HLH vs. PTA	0.0036
HLH vs. PS/PA	0.0115
HLH vs. DORV	0.0368
HLH vs. SD	0.0005
HLH vs. TAP	0.0007
HLH vs. PDA	0.0190
HLH vs. Others	0.0082
TOF vs. LVOTO	> 0.9999
TOF vs. RVOTO	> 0.9999
TOF vs. TGA	> 0.9999
TOF vs. PTA	> 0.9999
TOF vs. PS/PA	> 0.9999
TOF vs. DORV	> 0.9999
TOF vs. SD	> 0.9999
TOF vs. TAP	> 0.9999
TOF vs. PDA	> 0.9999
TOF vs. Others	> 0.9999
LVOTO vs. RVOTO	> 0.9999
LVOTO vs. TGA	> 0.9999
LVOTO vs. PTA	> 0.9999
LVOTO vs. PS/PA	> 0.9999
LVOTO vs. DORV	> 0.9999
LVOTO vs. SD	> 0.9999
LVOTO vs. TAP	> 0.9999
LVOTO vs. PDA	> 0.9999
LVOTO vs. Others	> 0.9999
RVOTO vs. TGA	> 0.9999

RVOTO vs. PTA	> 0.9999
RVOTO vs. PS/PA	> 0.9999
RVOTO vs. DORV	> 0.9999
RVOTO vs. SD	> 0.9999
RVOTO vs. TAP	> 0.9999
RVOTO vs. PDA	> 0.9999
RVOTO vs. Others	> 0.9999
TGA vs. PTA	> 0.9999
TGA vs. PS/PA	> 0.9999
TGA vs. DORV	> 0.9999
TGA vs. SD	> 0.9999
TGA vs. TAP	> 0.9999
TGA vs. PDA	> 0.9999
TGA vs. Others	> 0.9999
PTA vs. PS/PA	> 0.9999
PTA vs. DORV	> 0.9999
PTA vs. SD	> 0.9999
PTA vs. TAP	> 0.9999
PTA vs. PDA	> 0.9999
PTA vs. Others	> 0.9999
PS/PA vs. DORV	> 0.9999
PS/PA vs. SD	> 0.9999
PS/PA vs. TAP	> 0.9999
PS/PA vs. PDA	> 0.9999
PS/PA vs. Others	> 0.9999
DORV vs. SD	> 0.9999
DORV vs. TAP	> 0.9999
DORV vs. PDA	> 0.9999
DORV vs. Others	> 0.9999
SD vs. TAP	> 0.9999
SD vs. PDA	> 0.9999
SD vs. Others	> 0.9999
TAP vs. PDA	> 0.9999
TAP vs. Others	> 0.9999
PDA vs. Others	> 0.9999

The table lists p values from an ANOVA with a post-hoc Dunn’s test for multiple comparisons between patients with no applicable disease (NAD), hypoplastic left heart (HLH), Tetralogy of Fallot (TOF), left ventricular outflow tract obstruction (LVOTO), right ventricular outflow tract obstruction (RVOTO), transposition of the great arteries (TGA), Persistent truncus arteriosus (PTA), pulmonary stenoses or atresia (PS/PA), double outlet right ventricle (DORV), septal defects (SD), thoracic aortic pathologies (TAP), persistent ductus arteriosus (PDA), and others.

Supplementary Table S2: Diagnostic cross tables.

LVM vs. surgical approach	All patients		HLH-subgroup	
	Biventricular	Univentricular	Biventricular	Univentricular
LVM > 33.9 g/m^2	93	5	2	0
LVM < 33.9 g/m^2	20	14	2	10

Cross tables listing the patients with an LVM above or below the threshold of 33.9 g/m^2 along with the received surgical management for the entire patient population and the subgroup of HLH patients.
